# Revisiting the putative role of heme as a trigger of inflammation

**DOI:** 10.1002/prp2.392

**Published:** 2018-03-30

**Authors:** Florence Vallelian, Christian A. Schaer, Jeremy W. Deuel, Giada Ingoglia, Rok Humar, Paul W. Buehler, Dominik J. Schaer

**Affiliations:** ^1^ Division of Internal Medicine University of Zurich Zurich Switzerland; ^2^ Center of Biologics Evaluation and Research (CBER) FDA Silver Spring MD USA

**Keywords:** heme, hemoglobin, inflammation, innate immunity, protoporphyrin IX, sickle cell disease

## Abstract

Activation of the innate immune system by free heme has been proposed as one of the principal consequences of cell‐free hemoglobin (Hb) exposure. Nonetheless, in the absence of infection, heme exposures within a hematoma, during hemolysis, or upon systemic administration of Hb (eg, as a Hb‐based oxygen carrier) are typically not accompanied by uncontrolled inflammation, challenging the assumption that heme is a major proinflammatory mediator in vivo. Because of its hydrophobic nature, heme liberated from oxidized hemoglobin is rapidly transferred to alternative protein‐binding sites (eg, albumin) or to hydrophobic lipid compartments minimizing protein‐free heme under in vivo equilibrium conditions. We demonstrate that the capacity of heme to activate human neutrophil granulocytes strictly depends on the availability of non protein‐associated heme. In human endothelial cells as well as in mouse macrophage cell cultures and in mouse models of local and systemic heme exposure, protein‐associated heme or Hb do not induce inflammatory gene expression over a broad range of exposure conditions. Only experiments in protein‐free culture medium demonstrated a weak capacity of heme‐solutions to induce toll‐like receptor‐(TLR4) dependent TNF‐alpha expression in macrophages. Our data suggests that the equilibrium‐state of free and protein‐associated heme critically determines the proinflammatory capacity of the metallo‐porphyrin. Based on these data it appears unlikely that inflammation‐promoting equilibrium conditions could ever occur in vivo.

**Abbreviations**
TLR4: toll‐like receptor‐4
DAMPs: Damage‐associated molecular patterns
RBCs: red blood cells
Hb: hemoglobin
PHZ: phenylhydrazine hydrochloride
HUVECs: heme exposure in endothelial cells

## 1 INTRODUCTION

Damage‐associated molecular patterns (DAMPs) signal a disruption in tissue homeostasis to the immune system, initiating and amplifying inflammatory and regenerative responses.^1^ Over the last several years, an increasing number of reports have suggested that Hb‐derived heme could be such an endogenous danger signal, triggering inflammation whenever red blood cells (RBCs) are destroyed and Hb released into extracellular spaces.^2^, ^3^

Cell‐free Hb is considered an amplifier of disease in a range of pathologies.^2^ The most archetypical condition associated with extravascular Hb and heme release occurs when RBCs extravasate after a tissue trauma resulting in blood vessel injury and RBC leakage, causing either a hematoma or a contusion. Over time, these extravascular RBCs break down, releasing Hb and other cellular components. In contrast, systemic release of free Hb occurs during intravascular hemolysis, such as sickle cell disease or transfusion‐associated hemolysis.^4^ Extensive clinical experience suggests that hematomas, contusions, and systemic hemolysis are not usually associated with an exaggerated proinflammatory response. In contrast, cases of simple contusions presenting with cardinal signs of inflammation like edema, redness, and pain would raise concerns of an infection.^5^ Therefore, it would be more intuitive to assume that the accumulation of Hb in damaged tissues promotes noninflammatory or even anti‐inflammatory instead of proinflammatory effects. However, paradoxical observations have been reported in the literature over the last decade. Several studies have described proinflammatory signaling activities of heme via the activation of pattern recognition receptors such as TLR4 or the inflammasome signaling pathway in leukocytes and endothelial cells.^3^, ^6^, ^7^, ^8^, ^9^, ^10^, ^11^, ^12^, ^13^

An essential caveat of these opposing hypotheses regarding the function of heme in the regulation of inflammation may be determined by its macromolecular associations. Heme is a lipophilic molecule, which forms (mu‐oxo) dimers, oligomers, and larger aggregates in aqueous solutions.^14^, ^15^, ^16^ Therefore, heme exists almost exclusively bound within a hemoprotein (eg, Hb) in biological environments. Under certain conditions, such as after the oxidation of Hb to ferric metHb (HbFe^3+^), the association of heme with the hemoprotein (eg, globin) weakens.^17^, ^18^ However, even under these conditions, the binding of the “released” metallo‐porphyrin by other macromolecules such as albumin or lipids keeps the pool of “free” heme low.^14^ By this mechanism, free heme accumulation in vivo remains orders of magnitude below the conditions under which protein‐free heme‐solutions have been tested for proinflammatory activities in vitro.

Here, we present data obtained from several in vitro and in vivo models that support the more intuitive hypothesis that protein‐associated heme originating from RBC degradation does not induce inflammation.

## 2 METHODS

### 2.1 Heme preparation

#### 2.1.1 Preparation of heme‐albumin

Hemin (65 mg; Sigma 51280) was dissolved in 10 mL NaOH (100 mmol·L^−1^) at 37°C, and then 15 mL of 20% human serum‐albumin (CSL Behring AG) was added. After 1 hour of incubation at 37°C, the pH of the solution was adjusted to pH 7.8 using phosphoric acid. The 4 mmol·L^−1^ heme‐albumin solution was sterile‐filtered (0.22 μm) and used immediately.

#### 2.1.2 Preparation of heme‐NaOH

Heme was dissolved in 10 mL NaOH (100 mmol·L^−1^) at 37°C. The pH of the solution was adjusted to pH 7.8 using phosphoric acid. The solution was sterile‐filtered (0.22 μm) and used immediately. The purified Hb solutions that we used were described previously. metHb (ferric) was generated by incubating oxyHb (ferrous) with a 5 × molar excess of K3[Fe(CN)6] at room temperature for 10 minutes, followed by purification on a PD‐10 column (GE Healthcare). Hemoglobin concentrations and oxidation states were determined by spectral deconvolution, as described.^19^ Hemoprotein and heme concentrations are indicated as molar concentrations of heme.

### 2.2 Quantification of free heme by stopped‐flow spectrophotometry

Heme or hemoproteins were diluted in PBS, pH 7.4, to a final concentration of 5 μmol·L^−1^ in a stopped‐flow cell. Transfer of heme to hemopexin (Hx) was measured at a 2× molar excess of Hx as a change in absorbance at 411 nm. Absorbance was measured every 12.5 ms with a stopped‐flow spectrophotometer (SX18MV; Applied Photophysics Ltd., Leatherhead, UK). Each experiment was conducted in triplicate. Absorbance data were fitted to a three‐phase kinetics model to define heme fractions with fast, intermediate, and slow heme‐hemopexin transfer using the R statistical software.

### 2.3 Cell culture

#### 2.3.1 Endothelial cell culture

Human umbilical vein endothelial cells (HUVECs) were obtained from Lonza (Switzerland) and cultured in EGM medium (Lonza) under standard conditions.

#### 2.3.2 Macrophage differentiation and stimulation

Mouse bone marrow‐derived macrophages (BMDMs) were expanded and differentiated in RPMI medium supplemented with 10% FCS in the presence of recombinant mouse M‐CSF (Peprotech) for 7 days. At the end of the culture period, 100% of adherent cells were positive for F4/80 antigen.

#### 2.3.3 Neutrophil stimulation and flow cytometry

Fresh human donated blood was purchased from the Swiss Red Cross (SRK Blutspendedienst, Schlieren). The buffy coat was harvested in DPBS MgCl_2_/CaCL_2_ (Gibco #14040) containing 500 IE/mL heparin by washing three times with DPBS MgCL_2_/CaCL2 and centrifuging at 400*g* for 10 minutes at 4°C. After the final wash, the supernatant was aspirated, and the cells were stimulated with heme at 37°C. Cells were put on ice immediately, and after centrifugation at 400*g* for 10 minutes at 4°, cells were stained with CD15‐FITC antibody (Ab; Miltenyi Biotec #130‐081‐101), APC‐CD14 (BD Biosciences #555399), and PE‐CD62L (BD Biosciences #341012). Erythrocytes were lyzed with lyzing buffer (8.29 g ammonium chloride, 1 g potassium hydrogen carbonate, 0.037 g Na‐EDTA per L) at room temperature for 1 minute. Residual cells were analyzed by cytometry with a FACSCalibur (Becton Dickinson) flow cytometer. Neutrophil activation was measured by shedding of CD62L Ab (PE‐CD62L; BD Biosciences #341012). Data were analyzed using CellQuest Pro software, and logistic regression was performed with the R statistical software.

### 2.4 ATP measurement and metabolic flux analysis

A chemiluminescent assay was used to measure cellular ATP (CellTiter‐Glo Assay; Promega AG, Dubendorf, Switzerland). Experiments were performed in 96‐well plates, and luminescence was measured with an Infinite M200‐Pro plate reader (Tecan Group Ltd, Männedorf, Switzerland).

Mitochondrial function (oxygen consumption rate) and glycolysis (acidification rate) were measured with confluent monolayers of HUVECs with a Seahorse XFp extracellular flux analyzer and the Cell Mito Stress Kit (Seahorse Bioscience) according to the instructions provided by the manufacturer.

### 2.5 Western blotting

Samples were prepared with 5 μg of total protein per well in Lämmli buffer. The western blot protocol was previously described and included chemiluminescence detection of the secondary HRP‐labeled antibodies (Amersham, GE Healthcare, Glattbrugg, Switzerland) with the SuperSignal West Femto Maximum Sensitivity Substrate (Thermo Scientific AG). Images were optimized only by adjusting the brightness and contrast of the whole image using Photoshop Software (Adobe, Adobe Systems GmbH, Zürich, Switzerland). The primary antibodies were pAb anti‐Hmox1 antibody (Enzo Life Sciences #ADI‐SPA‐895) at a final concentration of 0.2 μg/L.

### 2.6 Gene expression analysis

#### 2.6.1 Microarray experiments and data analysis

Total RNA was isolated using the RNeasy Mini Kit according to the manufacturer's instructions, which included an on‐column DNA digestion step (RNase‐Free DNase Set; Qiagen Hombrechtikon, Switzerland). To ensure that only high‐quality RNA (RNA integrity number >7.0) was used for gene expression analysis, each RNA sample was checked on an RNA Nanochip with a Bioanalyzer 2100 (Agilent Technologies). RNA was quantified spectrophotometrically with a NanoDrop ND‐1000 spectrophotometer (NanoDrop Technologies, Wilmington, DE, USA). Fluorescently labeled cRNA was generated from 500 ng total RNA with the Quick Amp Labeling Kit (Agilent Technologies) according to the manufacturer's protocol, and differential gene expression profiling was performed by competitive dual‐color hybridization on whole mouse or human Genome Oligo Microarrays (mouse: G4846A, human: G4845A, 4 × 44 K, Agilent Technologies). Array slides were XDR‐scanned and analyzed with Feature Extraction Software Version 10.7.3.1 (Agilent Technologies). Statistical analysis and visualization was performed with JMP Genomics 7.0 (SAS Institute, Boeblingen, Germany). Full gene array data were submitted to the gene expression omnibus.

#### 2.6.2 Real‐time PCR

Reverse transcription was performed with TaqMan reverse transcription reagents (Life Technologies, Basel, Switzerland). Real‐time PCR was performed using the TaqMan Fast Advanced Master mix and TaqMan gene expression assays (Life Technologies) with FAM‐labeled probes for the following targets: IL‐6 (Mm00446190_m1), TNF‐alpha (Mm00443258_m1), and *Hprt* (Mm01545399_m1_m1). Relative mRNA levels were calculated by the 7500 Fast System Sequence Detection Software Version 1.4 (Applied Biosystems) after normalization of each experimental sample to *Hprt* levels.

### 2.7 Bio‐Plex cytokine assays

Concentrations of TNF‐alpha, IL‐6, and IL‐8 were determined using Bio‐Plex Cytokine Assays (Bio‐Rad). The assays were analyzed with a Bio‐Plex 200 system (Bio‐Rad). Results were analyzed using Bio‐Plex Data Pro software (Bio‐Rad).

### 2.8 VCAM1 cell surface expression

HUVECs were prestimulated with the indicated hemoprotein for 4 hour before stimulation with IL‐1b (100 U/mL) for an additional 4 hour. Cells were fixed with 3.7% formaldehyde in PBS and permeabilized with 0.1% Triton‐X in PBS. Cells were blocked with LI‐COR Odyssey Blocking Buffer (#P/N 927) and incubated with anti‐hVCAM‐1 (#BBA19, 1:8000, RnDsystem). Cells were washed three times with 0.1% Tween‐20 in PBS and incubated with IRDye^®^ 800CW donkey anti‐goat (1 μg/mL; LI‐COR #926‐32214) and CellTag™ 700 stain (0.1 μmol·L^−1^; LI‐COR #P/N 926‐41090) or DRAQ5™ (0.5 μmol·L^−1^; Cell Signaling Technologies #4084S). Cells were washed again three times and analyzed with an Odyssey infrared imager from LI‐COR and Odyssey software.

### 2.9 Animal experiments

Wild‐type C57Bl/6 mice were obtained from Charles River (Wilmington, MA, USA). BMDM were also generated from *Tlr4*‐, *Myd88*‐, and *Trif*‐deficient mice Charles River (Wilmington, MA, USA). Heme oxygenase (Hmox1)‐deficient macrophages were generated from B6.Cg‐Tg(UBC‐cre/ERT2)1Ejb/J x B6.129S‐Hmox1 mice.

#### 2.9.1 Matrigel plug assay

8‐week‐old C57Bl/6 mice were injected subcutaneously with 0.2 mL of Matrigel (Corning) containing 1 mmol·L^−1^ heme‐albumin or LPS (1 μg/mL). The injected Matrigel rapidly formed a single, solid gel plug. After 5 days the Matrigel plug was removed, fixed in formalin, and embedded in paraffin. Sections (3–5 μm thick) were prepared, mounted on glass slides, deparaffinized in xylene, rehydrated through graded alcohols, and stained with hematoxylin and eosin (HE) and Abs against F4/80 (Invitrogen MA5‐16363 poly R6), Lys6G (Biolegend 127602), and iNOS (EMD Millipore, ABN26). All slides were scanned using a digital slide scanner (NanoZoomer‐XR C12000, Hamamatsu, Japan), images were taken using NDP.view2 software (Hamamatsu).

#### 2.9.2 Laser microdissection

Freshly removed Matrigel plugs were embedded in OTC and frozen in liquid‐nitrogen‐cooled methanol. Cryosections (8 μm) were mounted on polyethylene naphthalate (PEN) membrane frame glass slides (LCM0522, ThermoFisher Scientific). PEN membrane slides were gradually hydrated to water/ethanol (1:1), stained with 4% cresyl violet (LCM Staining Kit, AM1935, Ambion), washed in water/ethanol, dehydrated to 100% ethanol, and cleared with 100% xylene. RNase contamination was avoided by cleaning surfaces and tools with RNase Zap^®^ solution. Regions of interest of sectioned Matrigel plugs were outlined manually and tagged digitally using an Arcturus XT™ Microdissection System equipped with a Nikon TE2000U brightfield microscope, cut by a digitally guided solid state UV laser, and glued to CapSure Micro LCM caps (A30153, ThermoFisher Scientific) by solid state infrared laser pulses. Film on caps containing specific biological material was removed by sterile forceps, transferred to 20 μL RT buffer, and frozen in dry ice before RNA isolation by iScript Reverse Transcription Supermix (Cat. 170‐8842, BioRad).

#### 2.9.3 PHZ‐induced systemic hemolysis

C57Bl/6 mice were treated intraperitoneally (i.p.) with 90 mg/kg phenylhydrazine hydrochloride (PHZ). Livers were collected, and macrophages were purified from cell suspensions by F4/80‐conjugated microbeads and magnetic cell separation columns (Miltenyi) according to the manufacturer's protocol (Miltenyi). The purity of isolated cells was confirmed by FACS and was always >80%. The concentration of heme was quantified in F4/80^+^ macrophages using the oxalic acid method of Sassa et al. 20 using oxalic acid from Sigma‐Aldrich.

### 2.10 Study approval

All experimental protocols were reviewed and approved by the Veterinary Office of the canton of Zürich. All animals were maintained at the animal facility of the University of Zurich and were treated in accordance with guidelines of the Swiss federal Veterinary Office.

## 3 RESULTS

### 3.1 Solution characteristics determine neutrophil activation by free heme

**Figure 1:**
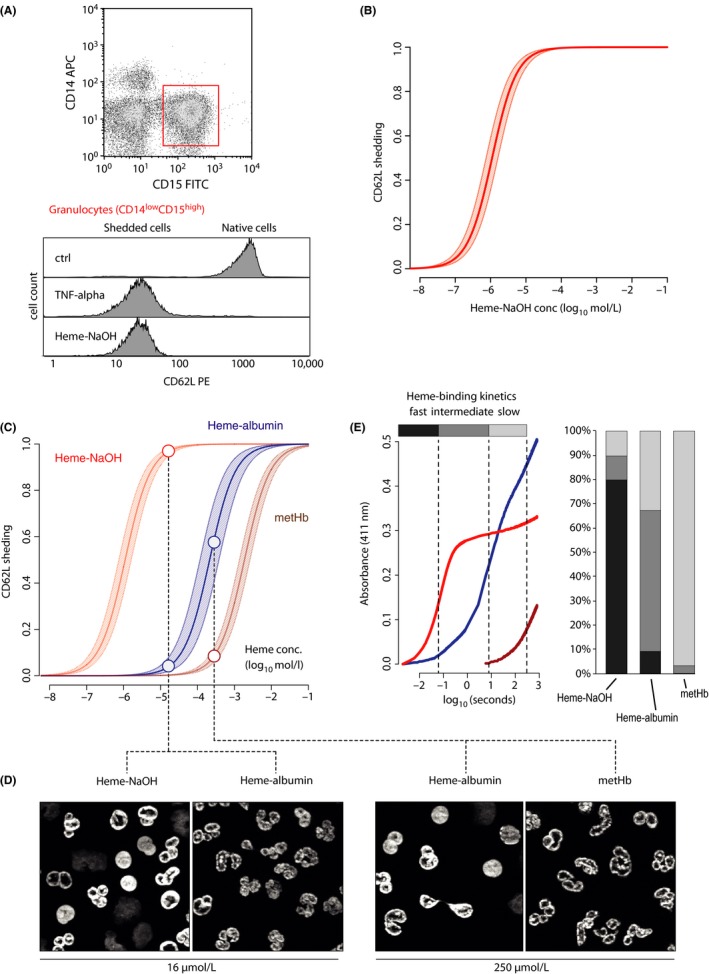
Effect of heme solution characteristics on granulocyte activation. (A) Granulocytes were identified by flow‐cytometry as the CD14^low^ CD15^high^ population. Surface expression of CD62L was analyzed in response to a 30‐minute exposure to TNF‐alpha or NaOH‐dissolved heme. Reduction of CD62L‐signal indicates shedding. (B) Dose–effect relationship of NaOH‐dissolved heme concentration and CD62L‐shedding (fraction of CD62L‐negative granulocytes). Shown is the mean ± SD of the logistic regression of data obtained from dose–response experiments performed with blood from five healthy donors (10 heme concentrations were tested per experiment). (C) CD62L shedding induced by various heme formulations: protein‐free heme (NaOH‐dissolved heme) in red, heme‐albumin in blue, and metHb in brown. (D) DNA (DAPI) stains of nuclei of neutrophil granulocytes exposed to heme at the concentrations indicated by the dots in Figure 1C. While at 16 μmol·L^−1^, heme‐albumin induces virtually no nuclear condensation, NaOH‐dissolved heme exerts an apoptotic effect. The same can be observed at much higher concentrations (250 μmol·L^−1^) when comparing heme‐albumin to metHb. (E) Stopped‐flow experiments of heme to hemopexin transfer. Heme transfer to hemopexin can be considered a sensitive method for detecting free (ie, solubilized) heme. The fast fraction represents free heme, the intermediate fraction represents heme only loosely bound to proteins or present in aggregates, and the slow fraction represents heme tightly bound to proteins. The distribution of heme to these three compartments is visualized by the bar plots and is distinct for all three heme formulations

**Figure 2:**
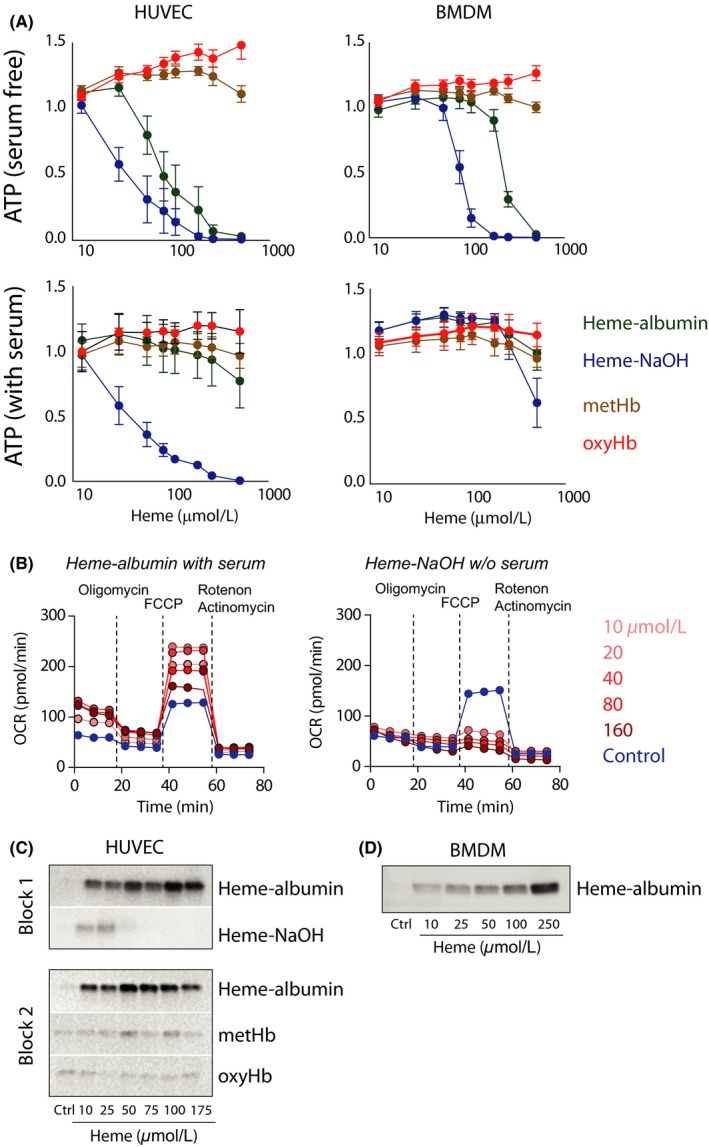
Effect of heme solution characteristics on macrophage and endothelial cell viability, metabolic activity, and HMOX1 expression. (A) Cellular ATP as a fraction of control values in HUVECs and mouse BMDMs after treatment with protein‐free heme (NaOH), heme‐albumin, metHb, or oxyHb for 8 hours. Identical experiments were performed in cell culture medium with or without serum (2% FCS for HUVECs and 10% FCS for BMDMs). Data represent the mean ± SEM of six biological replicates. (B) Extracellular flux analysis of heme‐exposed HUVECs. Oxygen consumption rate (OCR) was measured in HUVECs after exposure to a range of concentrations of heme‐albumin (in the presence of 2% FCS) or NaOH‐dissolved heme in the absence of serum. (C) Western blot analysis of HMOX1 protein in HUVECs after exposure to a range of concentrations of heme‐albumin or NaOH‐dissolved heme (block 1) or heme‐albumin, metHb, or oxyHb (block 2). Proteins in the same block were run on the same gel. (D) Western blot analysis of HMOX1 protein in mouse BMDMs after exposure to a range of concentrations of heme‐albumin

Neutrophil granulocytes are known to be very sensitive to exposure to diverse inflammatory stimuli that ultimately trigger a conserved activation response, including the shedding of cell surface E‐selectin (SELE). Exposure of washed human peripheral blood leukocytes to protein‐free crystalline heme that has been dissolved in NaOH (100 mmol·L^−1^) with subsequent adjustment to pH 7.8 resulted in a drastic reduction in cell surface CD62L expression on CD14^low^CD15^high^ neutrophil granulocytes after 30 minutes of exposure (Figure 1A). This effect was comparable to the effect induced by TNF‐alpha. The effect of heme was dose‐dependent with 100% shedding at micromolar concentrations, as demonstrated by the logistic regression model shown in Figure 1B (aggregated data from 5 healthy blood donors with heme exposure assessed at 10 concentrations from 10^−8^ to 10^−1^ mol/L, see also Figure S1). In the next set of experiments, we compared the neutrophil‐activating effect of NaOH‐dissolved heme with the effects of more physiological protein‐associated heme, namely, heme‐albumin and metHb (HbFe^3+^) (Figure 2C). Protein association of heme with albumin or Hb shifted the dose‐response curve for neutrophil CD62L shedding to the right by several orders of magnitude. By modeling the equilibrium concentration in the experiments performed with heme‐albumin we could show that the free heme is the active neutrophil‐stimulating component, which is inactivated by protein association (Figure S2). With metHb, significant shedding was only observed at heme concentrations above 1 mmol·L^−1^, which corresponds to >16 mg/mL metHb. The shedding of CD62L correlated with the nuclear condensation of granulocytes observed 2 hour after stimulation (Figure 1D). It is important to note that these experiments were performed in serum‐ and protein‐free conditions, meaning that there were no alternative heme‐association sites available in the incubation medium.

To estimate concentrations of free heme or quasi‐free heme (eg loosely protein‐associated) in the different experimental heme solutions, we used stopped‐flow spectrophotometry to measure the fraction of heme that was available for rapid hemopexin binding (Figure 1E). In NaOH‐dissolved heme, which induced CD62L shedding at micromolar concentrations, approximately 80% of the heme demonstrated an ultra‐fast binding pattern (heme‐hemopexin complex formation completed within <100 milliseconds). In contrast, the ultra‐fast hemopexin‐binding fraction of heme was reduced to less than 10% in heme‐albumin. In solutions of metHb, the proportion of free heme available for ultra‐rapid hemopexin binding was found to be very low. These observations suggest that in any physiological scenario in which heme might be present in extracellular spaces as a component of a natural hemoprotein, the concentration of free or quasi‐free heme can be expected to be very low, possibly below the minimum range required to trigger granulocyte activation and other innate immunity responses.

### 3.2 Effects of heme solution characteristics on heme‐triggered heme oxygenase induction, cell viability, and metabolic disruption

Our initial experiments suggested that artificial preparations of protein‐free heme could trigger cellular responses that might not be reproduced by physiological sources of heme in vivo. Before we aimed to characterize in more detail the putative inflammatory properties of the more physiological protein‐associated heme, we defined how protein‐free heme and protein‐associated heme may affect cellular viability, metabolism, and markers of heme exposure in endothelial cells (HUVECs) and mouse bone marrow‐derived macrophages (BMDMs). In serum‐free conditions, NaOH‐dissolved heme (final pH 7.8) reduced cellular ATP in both cell types in a dose‐dependent manner after 8 hours of exposure (Figure 2A). In contrast, less ATP depletion was observed during exposure to heme‐albumin, and no effect could be detected when either cell type was exposed to metHb or oxyHb at concentrations of up to 500 μmol·L^−1^, even in the absence of serum. When the identical experiments were performed in the presence of serum (2% FCS in endothelial cells and 10% FCS in macrophage experiments), the heme‐induced effects were further reduced or completely eliminated.

We also confirmed the critical role of protein association in cellular heme effects in a metabolic experiment using a Seahorse extracellular flux analyzer (Figure 2B). This experiment demonstrated that under serum‐free conditions, 4 hours of exposure to NaOH‐dissolved heme induced complete deterioration of mitochondrial respiration at concentrations as low as 10 μmol·L^−1^, while exposure to albumin‐associated heme in the presence of 2% FCS stimulated respiratory capacity to levels above baseline.

The expression of heme oxygenase (HMOX1) can be considered a direct signal of intracellular heme translocation in healthy cells. In endothelial cells, we found that, in the absence of serum, heme‐albumin triggered a dose‐dependent HMOX1 protein expression signal in an exposure range of 0–175 μmol·L^−1^. In contrast, exposure to NaOH‐dissolved heme resulted in virtually no signal, presumably as a result of the strong toxicity of the treatment. Heme translocation with subsequent HMOX1 induction was very limited upon treatment with metHb or oxyHb (Figure 2C). This observation is consistent with the very low free heme fraction in these solutions. Heme‐albumin is also a robust inducer of dose‐dependent HMOX1 expression in BMDMs (Figure 2D).

### 3.3 Protein‐associated heme does not trigger inflammation in human endothelial cells

Thus far, we have determined that protein association dramatically reduces free heme availability and limits the range of heme‐inducible biological responses. As an experimental model, exposure to heme‐albumin in the presence of low concentrations of serum may mimic the most severe conditions of heme exposure during tissue trauma or hemolysis in vivo. We therefore explored whether exposure of endothelial cells and macrophages to heme‐albumin or to the natural heme proteins, oxyHb and metHb, could trigger inflammatory responses, which would justify the classification of heme as a bona fide DAMP.

**Figure 3:**
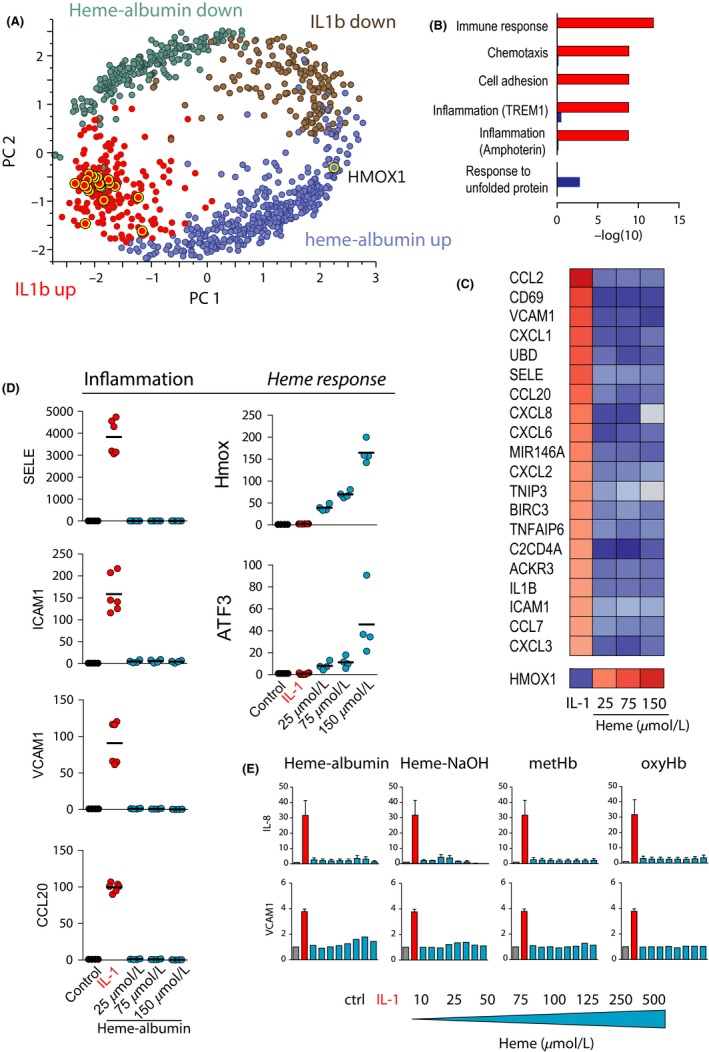
Effect of hemoproteins on human endothelial cells. (A‐C) Gene array analysis of gene expression (mRNA) changes in HUVECs treated for 8 hours with IL‐1b or heme‐albumin (25 μmol·L^−1^, 75 μmol·L^−1^ and 150 μmol·L^−1^) (n = 4 experiments for each heme treatment, n = 6 experiments for IL‐1b). (A) Principal component analysis (PCA) of the standardized least‐square means of all genes that were regulated by more than 2‐fold (FDR < 0.01). Each expressed gene is represented as a dot and color‐coded for its expression category (up‐ or downregulation by heme or IL‐1b). The yellow enhancement indicates those genes that are further analyzed in (C). (B) Metacore enrichment analysis for gene functions assigned to the IL‐1b‐upregulated genes (red bars) and heme‐upregulated genes (blue bars) (regulation cut‐off 4‐fold, FDR < 0.01). (C) Heatmap of the expression level of the 20 most IL‐1b upregulated genes in comparison to the expression level in heme‐albumin treated HUVEC samples. The selected transcripts are color‐coded (red: high expression; blue: low expression). (D) Relative mRNA expression of proinflammatory molecules (inflammation) and oxidative stress (heme response) genes in HUVECs in response to IL‐1b or increasing concentrations of heme‐albumin. Data represent the mean ± SD of six replicates. (E) Concentration of IL‐8 and VCAM1 in the cell culture supernatant of HUVECs in response to increasing concentrations of NaOH‐dissolved heme, heme‐albumin, metHb, and oxyHb over an exposure range of 10 to 500 μmol·L^−1^ for 6 hours. Experiments were performed in complete cell culture medium containing 2% FCS. Data represent the mean ± SD of six replicates

Principal component analysis (Figure 3A) of gene array expression data obtained from RNA extracted from nonstimulated HUVECs and from HUVECs exposed to either 150 μmol·L^−1^ heme‐albumin or IL‐1b revealed a strongly dichotomous response pattern triggered by the two treatments. The group of genes regulated by the archetypical proinflammatory stimulus IL‐1b displayed virtually no overlap with the genes that were regulated by heme‐albumin. An enrichment analysis for gene functions revealed that IL‐1b‐regulated genes (*IL‐1b up*, red label) belonged to categories such as immune response, chemotaxis, cell adhesion, and inflammation (Figure 3B). In contrast, no significant inflammation‐related enrichment was observed for the heme‐albumin‐triggered response genes (*heme‐up*, blue label), which were more characteristic of categories such as response to unfolded proteins. More specifically, as shown in Figure 3C, the top IL‐1b‐induced genes were not induced by heme‐albumin at 25, 75, or 150 μmol·L^−1^. Heme‐albumin, in contrast, induced HMOX1 in a dose‐dependent manner. The strictly dichotomous responses triggered by the archetypical inflammatory stimulator IL‐1b and by heme‐albumin were confirmed by RT‐PCR analysis of the expression of *SELE*,* ICAM1*,* VCAM1*,* CCL20*,* HMOX1*, and the oxidative response gene *ATF3* (Figure 3D). In the presence of 2% FCS, none of the initially explored heme compounds (NaOH‐dissolved heme, heme‐albumin, metHb, or oxyHb) induced appreciable secretion of IL‐8 or upregulation of cell‐surface VCAM1 over an exposure range of 10–500 μmol·L^−1^ for 6 hours (Figure 3E).

### 3.4 Protein‐associated heme does not trigger inflammation in mouse macrophages

**Figure 4:**
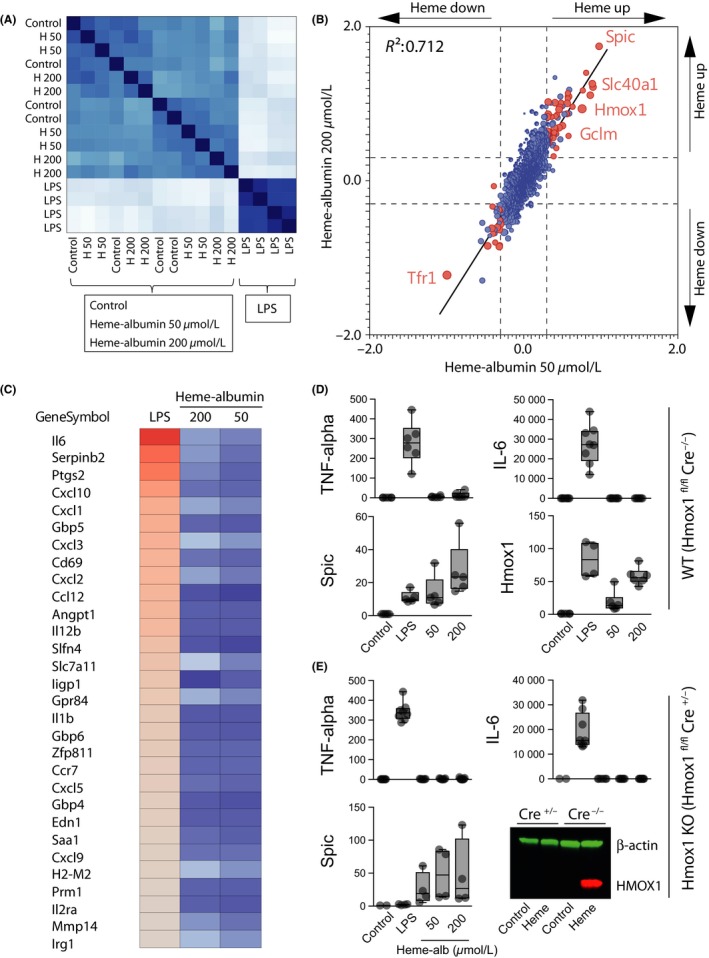
Effect of hemoproteins on bone marrow‐derived mouse macrophages. (A‐C) Gene array analysis of gene expression (mRNA) changes in mouse BMDMs treated for 8 hours with LPS (10 ng/mL) or heme‐albumin (50 μmol·L^−1^ and 200 μmol·L^−1^) (n = 4 experiments per condition). (A) Cross‐correlation plot of gene expression data of all genes across all treatment conditions. The data show a clear separation of LPS‐treated samples from all other conditions. (B) Dot‐plot and correlation analysis of the gene expression data from BMDMs treated with 50 μmol·L^−1^ or 200 μmol·L^−1^ heme‐albumin (for 8 hours) relative to control cells. Genes that are significantly up‐ or downregulated by 50 μmol·L^−1^ heme are highlighted in red. Representative heme‐response genes are labeled. (C) Heatmap of mRNA expression data across exposure conditions for the most LPS‐upregulated genes (red: high expression; blue: low expression). (D) RT‐PCR analysis of mRNA expression in wild‐type mouse BMDMs (tamoxifen‐treated Hmox1 ^fl/fl^ Cre^−/−^) after exposure to the indicated stimuli for 8 hours. Data are presented relative to control mRNA levels and represent 6–8 independent experiments. (E) Identical analysis as in (D) with *Hmox1*‐knockout macrophages (tamoxifen‐treated Hmox1 ^fl/fl^ Cre^+/−^). Western blot demonstrates HMOX1 protein in wild‐type (Cre^−/−^) and knockout (Cre^+/−^) macrophages after heme treatment

In addition to endothelial cells, macrophages were considered another possible target of heme‐triggered proinflammatory activity. As with endothelial cells, we started the analysis of macrophage responses with a gene array study comparing changes in gene expression induced by 50 and 200 μmol·L^−1^ heme‐albumin and by LPS. Figure 4A shows the cross‐correlation analysis of all genes represented on the array. The results revealed a clear separation between the LPS‐treated samples and all other conditions, while the heme‐albumin‐treated samples were not separated from the control samples. Figure 4B shows a more detailed gene expression correlation analysis of the samples treated with low (50 μmol·L^−1^, n = 4) and high (200 μmol·L^−1^, n = 4) concentrations of heme‐albumin. The most significantly regulated genes at both concentrations were *Spic*, a macrophage‐restricted transcription factor with a role in red‐pulp macrophage differentiation; *Hmox1*, the iron exporter *Scl40A1* (ferroportin); and the antioxidant response gene *Gclm*. This analysis therefore does not provide evidence of a heme‐triggered inflammatory response. In addition, a more detailed analysis reveals no significant induction of LPS‐induced genes by heme‐albumin (Figure 4C). As shown in Figure 4D, RT‐PCR gene expression data for TNF‐alpha, IL‐6, *Spic,* and *Hmox1* from an independent set of experiments were consistent with these results. We have also repeated these studies, yielding identical results, with Hmox1‐deficient macrophages. These experiments exclude the possibility that exaggerated expression of Hmox1 could explain the absence of inflammation (Figure 4E).

**Figure 5:**
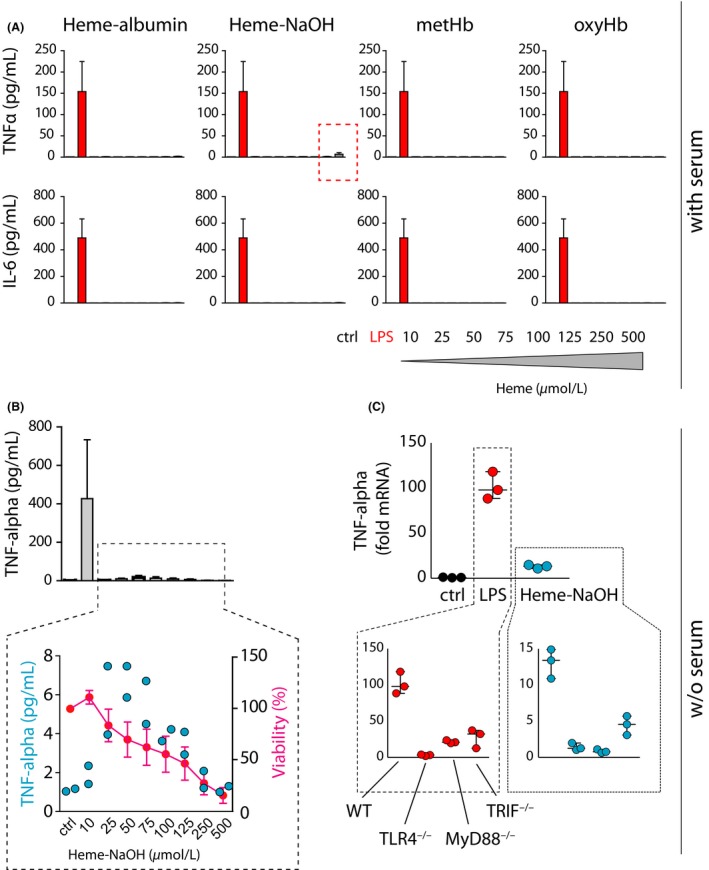
Heme‐triggered inflammation under protein‐free conditions. (A) Concentrations of TNF‐alpha (pg/mL) and IL‐6 (pg/mL) in the cell culture supernatant of mouse BMDM after treatment with a concentration gradient of heme‐albumin, NaOH‐dissolved heme, metHb, oxyHb, or LPS (10 ng/mL) for 18 hours. Experiments were performed in complete culture medium containing 10% FCS. (B) Concentration of TNF‐α in the cell culture supernatant of mouse BMDMs treated with NaOH‐dissolved heme or LPS in serum‐free cell culture medium for 18 hours. In parallel, cellular ATP was determined as a measure of viability and is expressed as a percentage of untreated cells. (C) TNF*‐*alpha mRNA expression in mouse BMDMs that were treated with NaOH‐dissolved heme (50 μmol·L^−1^) or LPS (10 ng/mL) for 4 hours in serum‐free cell culture medium. Identical experiments have been performed with BMDM from wild‐type, *Tlr4*‐knockout, *Myd88*‐knockout, or *Trif*‐knockout mice (plots in the dashed areas, note different scales of y‐axes for LPS and Heme‐NaOH stimulatd cells)

We also measured the secretion of the inflammatory mediators TNF‐alpha and IL‐6 from mouse BMDMs into the culture medium during treatment with NaOH‐dissolved heme, heme‐albumin, oxyHb, and metHb for 18 hours (culture medium contained 10% FCS). Again, compared to the archetypical proinflammatory stimulus LPS, we did not detect appreciable cytokine secretion under most conditions (Figure 5A). However, a weak TNF‐alpha signal appeared in macrophages that were treated with the highest concentration of NaOH‐dissolved heme (500 μmol·L^−1^). We therefore repeated this experiment in serum‐free medium and found the TNF‐alpha secretion response shifted to the left with a maximum signal at 50 μmol·L^−1^ heme (Figure 5B). At higher heme concentrations, TNF‐alpha secretion declined due to increasing toxicity, as indicated by a decline in intracellular ATP (shown in red). Further experiments using macrophages from different mouse knockout strains suggested that this proinflammatory heme response in the serum‐free model was a result of signaling via the TLR4–MyD88–TRIF pathway (Figure 5C). These experiments confirm a number of earlier reports and support the idea that certain solutions derived from purified/crystalline heme have the potential to induce weak TLR4‐mediated inflammatory responses in macrophages in the complete absence of plasma‐derived proteins.

**Figure 6:**
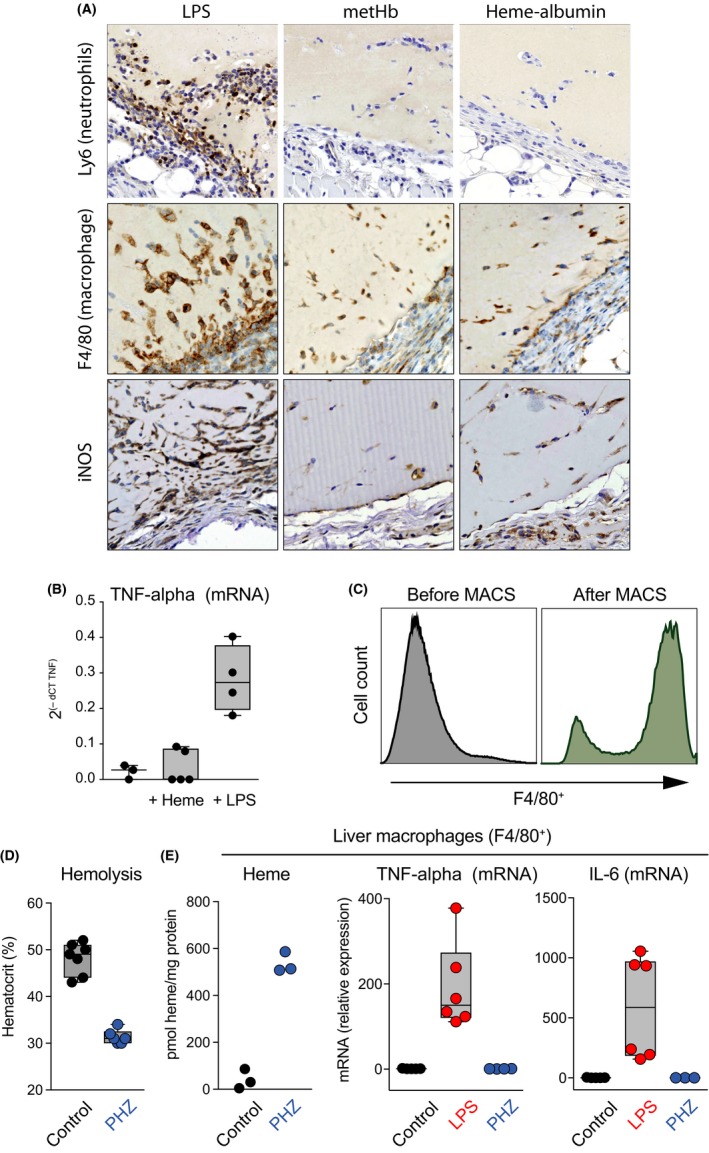
Effects of heme on inflammatory pathways in vivo. (A) Subcutaneous Matrigel plugs were explanted 5 days after implantation and stained for Ly6 (granulocytes), F4/80 (macrophages), and iNOS. Plugs were enriched with LPS (1 μg/mL), metHb (1 mmol·L^−1^), or heme‐albumin (1 mmol·L^−1^). (B) The cellular infiltrate from control, heme‐albumin and LPS enriched plugs were isolated at day 5 after injection by laser‐microdissection (LCM), and TNF *‐*alpha mRNA expression was analyzed by RT‐PCR. Relative expression levels were normalized for *HPRT* mRNA expression and are displayed as 2^−^ ^dCT^ ^(^ ^TNF^ ^−^ ^HPRT^ ^)^. (C) FACS analysis of F4/80 expression on liver cells before and after purification with F4/80‐conjugated magnetic beads. (D) Hematocrit levels of mice treated with saline (control) or phenylhydrazine (PHZ 90 mg/kg), determined 48 hours after treatment. (E) Analysis of heme content, TNF‐alpha mRNA expression, and IL‐6 mRNA expression in F4/80^+^ macrophages of the liver from control mice and mice treated with PHZ or LPS (0.5 mg/kg) for 4 hours

In our last set of studies, we aimed to explore whether heme could support a proinflammatory environment in vivo. In the first model, subcutaneous Matrigel^®^ plugs were injected bilaterally into the flanks of wild‐type C57Bl/6 mice. The plugs were recovered for analysis after 5 days. The plugs were enriched with either LPS (1 μg/mL), heme‐albumin (1 mmol·L^−1^), or metHb (1 mmol·L^−1^). After 5 days in situ, the LPS‐enriched plugs showed a cellular infiltrate rich in Ly6^+^ neutrophils and iNOS‐expressing F4/80^+^ macrophages. In contrast, the heme‐albumin and metHb‐enriched plugs were not different from control plugs, exhibiting few infiltrating macrophages (Figure 6A). We also measured TNF‐alpha mRNA expression of the cellular infiltrate in the different plugs by RT‐PCR (Figure 6B). While control and heme‐enriched plugs had minimal TNF‐alpha expression, significantly higher levels of TNF‐alpha mRNA were found in the LPS‐enriched plugs.

In the second model, we injected phenylhydrazine (PHZ) into mice to induce severe and acute hemolysis, with a drop in hematocrit from 48% to 31% within 48 hours (Figure 6D). This treatment was accompanied by drastic increases in heme concentrations in F4/80^+^ liver macrophages (Figure 6C). However, compared to treatment with LPS, systemic hemolysis did not provoke enhanced expression of TNF‐alpha mRNA or IL‐6 mRNA in F4/80^+^ Kupffer cells of the liver (Figure 6E).

## 4 DISCUSSION

The overall framework of Hb‐mediated disease is based on three predominant hypotheses, which link specific biochemical properties of Hb and heme with pathophysiological processes that can occasionally be observed in patients with hemolysis or at sites of local Hb accumulation.^2^ The first process links the nitric oxide (NO) reactivity of oxyHb to a cascade of cell‐free Hb extravasation, hemolysis‐associated NO‐depletion, and vasoconstriction.^21^, ^22^, ^23^ The second biochemical process relates to the ability of Hb to facilitate oxidative reactions outside of the reducing environment of the RBC, leading to the accumulation of ferric metHb(Fe^3+^) in tissue.^24^ In subsequent reactions, metHb or metHb‐derived heme participates in redox chain reactions that lead to the accumulation of modified lipids and proteins, as well as to heme degradation and to the release of free iron.^19^, ^25^ Ultimately, these processes can result in oxidative tissue injury, which may be accompanied by a tissue regenerative response or by secondary inflammation.^26^ The third process suggests that free heme could be recognized by innate immunity receptors as an endogenous DAMP, which may directly activate inflammatory signaling pathways in leukocytes and endothelial cells. This mechanism has been suggested to contribute to the chronic inflammatory state that is observed in sickle cell disease.^10^ Furthermore, various studies in sickle cell disease mouse models have demonstrated that the injection of heme solutions can trigger endothelial hyperactivation, leading to vasoocclusion or an acute chest syndrome‐like phenotype.^11^, ^27^ Purified heme was found to be an activator of TLR4 8, 11, 27 in some studies and of the inflammasome 9 in others, and these activities were considered to be the molecular mechanism behind the coexistence of inflammation and hemolysis.

Our current study focused on the third pathophysiological process and aimed to systematically address in different cell culture and animal models the putative proinflammatory activity of hemoproteins. The hypothesis that cell‐free Hb or Hb‐derived heme may function as a DAMP that signals tissue injury to the innate immune system, initiating an enhanced immune response, is appealing. However, clinical experience indicates that a sterile tissue injury with a hematoma, where significant free Hb and heme accumulation occurs, is usually not accompanied by an uncontrolled inflammatory response. We have considered the possibility that pathophysiological responses to heme could be related to its physicochemical properties and macromolecular interactions. Therefore, variation in heme preparations may explain the differing observations regarding its effects on the inflammatory system.

Using protein‐associated heme as a stimulus and in the presence of low concentrations of serum, we were not able to detect inflammatory responses in macrophages or endothelial cells, even during exposure to very high concentrations of heme (500 μmol·L^−1^). This was true not only for stimulation with the natural hemoproteins ferrous and ferric Hb, but also for albumin‐associated heme. The heme‐albumin solution that we used as a model for protein‐associated heme was a mixture of albumin and heme in a 1:4 molar ratio. This solution contained a significant fraction of heme that was only weakly associated with the protein. We do not know of any pathological condition that would lead to such a dramatic accumulation of loosely protein‐associated heme remaining available for interactions with inflammatory cells after Hb degradation. However, similar to Hb, heme‐albumin did not induce an inflammatory response in macrophages or endothelial cells in the presence of low concentrations of serum. To escalate the level of cellular heme exposure, we tested Hmox1‐deficient macrophages and found the same lack of an inflammatory response. The only consistent response that we observed to heme exposure included the activation of an oxidative stress response in endothelial cells at higher heme concentrations and the induction of a set of genes in mouse BMDMs that are characteristic of Hb‐iron‐handling macrophages in the spleen including the transcriptional repressor *Spic*,* Hmox1*, and ferroportin (*Slc40A1*).

Only under protein‐free conditions did we observe a limited heme‐induced TNF‐alpha response in cultured macrophages, which was triggered via signaling of the classical TLR4–MyD88–TRIF pathway of NF‐kB activation.^28^ However, even this response was small compared to the LPS response observed in the same cells, and it occurred only at the boundary of heme‐induced cytotoxicity. To further support the hypothesis that the extent and nature of macromolecular associations determine whether heme does or does not function as an activator of immune cells, we explored heme‐ and hemoprotein‐triggered activation of neutrophil granulocytes in washed peripheral whole blood cells. In the absence of serum, this model appears to be extremely sensitive for the study of the dose‐response relationships of heme‐triggered leukocyte activation.^6^, ^29^, ^30^ Our experiments demonstrated that as free heme concentrations decline with the increasing availability and strength of heme–protein associations (protein‐free heme ≪ heme‐albumin < metHb), neutrophil CD62L shedding disappears. Our studies were not designed to structurally characterize the neutrophil‐activating species in protein‐free heme solutions. However, in the absence of macromolecular associations, such as in an aqueous solution of purified iron protoporphyrin IX, aggregates of heme are known to form. We also found by dynamic light scattering that protein‐free aqueous heme solutions at neutral pH values contained aggregates with a particle size of up to 1000 nm (mean diameter, 320 nm; data not shown). Such aggregates, which were not detectable in protein‐associated heme solutions, are a strong candidate for an innate immunity receptor activator. Alternatively, the complete absence of other proteins may provide an environment for low‐affinity interactions of mono‐ or oligomeric heme with TLR4 or with components of the inflammasome in vitro.

The absence of a liver macrophage inflammatory response during severe intravascular hemolysis and the absence of inflammatory activation of invading macrophages in heme‐enriched Matrigel plugs substantiated our principal findings in two in vivo models.

Our in vitro and in vivo studies suggest that physiological heme is likely not a bona fide activator of inflammation. However, it might still be possible that heme or Hb could alter inflammation in more complex models of sequential priming and activation processes or as a synergistic agonists with inflammatory activators.^31^, ^32^ Heme could, for example, enhance priming of leukocytes for subsequent activation by classical inflammatory stimuli. As an example of a synergistic model it has been observed that Hb could enhance macrophage activation by multiple TLR agonists by a so far undefined mechanism.^33^

## DISCLOSURES

The authors have declared that no conflict of interest exists.

## AUTHOR CONTRIBUTIONS

FV designed the study, performed experiments, analyzed data, and wrote the paper; CAS performed experiments and analyzed data; JWD performed experiments and analyzed data; GI performed experiments; RH performed experiments; PWB designed the study and wrote the paper; DJS designed the study, performed experiments, analyzed data, and wrote the paper.
