# Supplementary material for: Revisiting the putative role of heme as a trigger of inflammation
Source: Pharmacol Res Perspect. 2018 Mar 30;6(2):e00392. doi: 10.1002/prp2.392 (PMC5878102; doi:10.1002/prp2.392)

Supplementary Figure 1

Logistic regression of FACS Data. Neutrophils were stimulated with heme (NaOH: heme dissolved in NaOH, Alb: albumin-associated heme, met: metHb) as described in the methods and results sections and the fraction of CD62L negative neutrophils was quantified (red dots). Using R statistical software version 3.4.1, a logistic regressions was fitted by using non-linear least squares (dark red line).

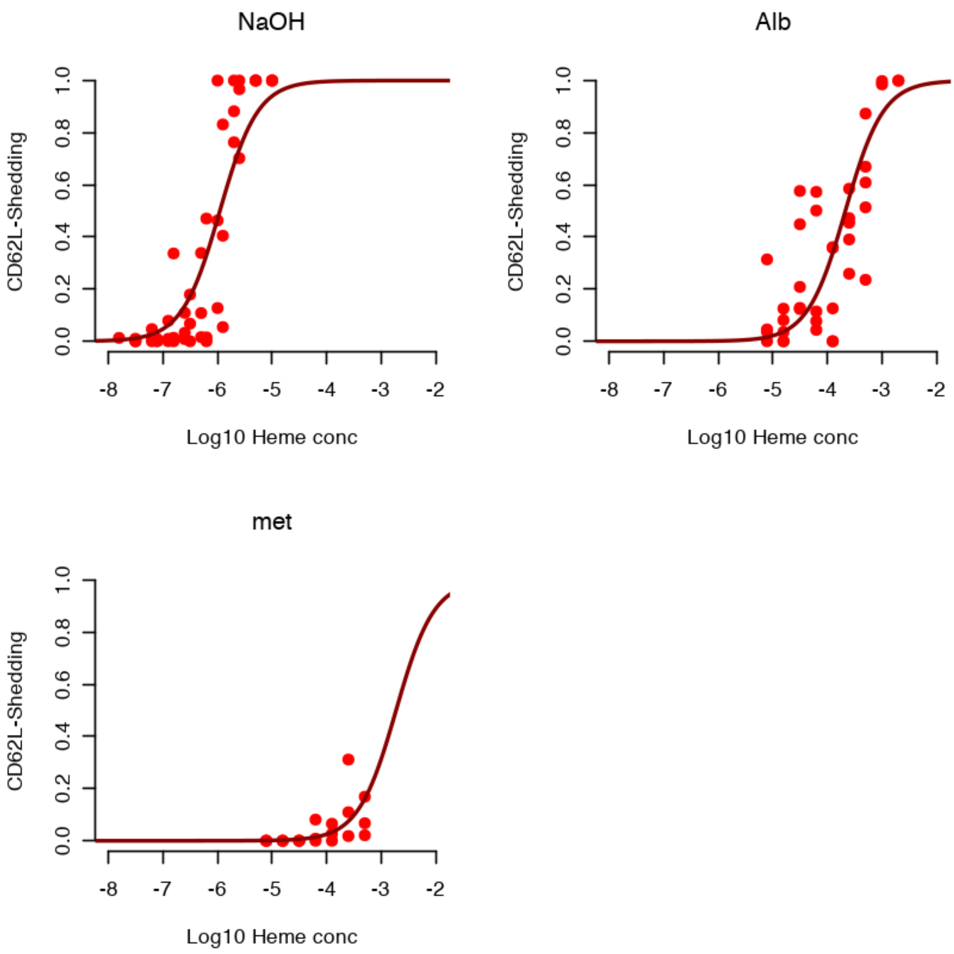

Supplementary Figure 2

ED50 of free and protein-associated heme in neutrophil CD62L-shedding experiments. The graph demonstrates the heme concentration at which 50% of neutrophils become CD62L negative after treatment. Data are derived from the logistic regression model shown in supplementary figure 1.

For albumin-associated heme (Alb), the equilibrium concentration of free heme in the experiment was estimated using the model obtained from the stopped flow experiments shown in Figure 1. The derived concentration of free heme(ED50) is indicated by the ⊠ symbol. It can easily be seen, that the fraction of free heme (⊠) is in the concentration range of NaOH-heme causing the same CD62L-shedding effect in the experiments performed in protein-free conditions. This data indicates that the unbound, free heme fraction, which is about 1/300th of the total heme, is the only active component in the experiment.

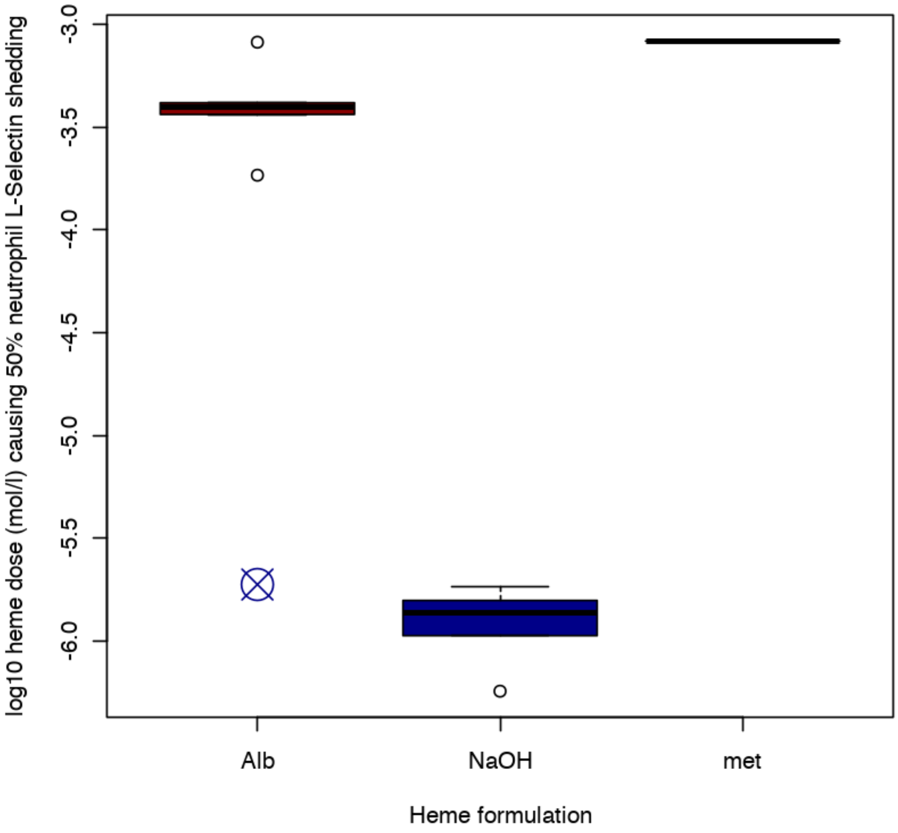

Supplement: Supplementary file 1 [file PRP2-6-e00392-s001.pdf]
